# Dysfunction in the Interaction of Information Between and Within the Bilateral Primary Sensory Cortex

**DOI:** 10.3389/fnagi.2022.862107

**Published:** 2022-04-08

**Authors:** Xiang-Xin Xing, Zhen-Zhen Ma, Jia-Jia Wu, Jie Ma, Yu-Jie Duan, Xu-Yun Hua, Mou-Xiong Zheng, Jian-Guang Xu

**Affiliations:** ^1^Department of Rehabilitation Medicine, Yueyang Hospital, Shanghai University of Traditional Chinese Medicine, Shanghai, China; ^2^School of Rehabilitation Science, Shanghai University of Traditional Chinese Medicine, Shanghai, China; ^3^Engineering Research Center of Traditional Chinese Medicine Intelligent Rehabilitation, Ministry of Education, Shanghai, China; ^4^Department of Rehabilitation Medicine, Longhua Hospital, Shanghai University of Traditional Chinese Medicine, Shanghai, China; ^5^Department of Traumatology and Orthopedics, Yueyang Hospital, Shanghai University of Traditional Chinese Medicine, Shanghai, China

**Keywords:** carpal tunnel syndrome, primary sensory cortex, effective connectivity, functional connectivity, voxel-mirrored homotopic connectivity, interhemispheric

## Abstract

**Background:**

Interhemispheric and intrahemispheric long-range synchronization and information communication are crucial features of functional integration between the bilateral hemispheres. Previous studies have demonstrated that disrupted functional connectivity (FC) exists in the bilateral hemispheres of patients with carpal tunnel syndrome (CTS), but they did not clearly clarify the phenomenon of central dysfunctional connectivity. This study aimed to further investigate the potential mechanism of the weakened connectivity of primary somatosensory cortex (S1) based on a precise template.

**Methods:**

Patients with CTS (*n* = 53) and healthy control subjects (HCs) (*n* = 23) participated and underwent resting-state functional magnetic resonance imaging (rs-fMRI) scanning. We used FC to investigate the statistical dependency of the whole brain, effective connectivity (EC) to analyze time-dependent effects, and voxel-mirrored homotopic connectivity (VMHC) to examine the coordination of FC, all of which were adopted to explore the change in interhemispheric and intrahemispheric S1.

**Results:**

Compared to the healthy controls, we significantly found a decreased strength of the two connectivities in the interhemispheric S1_*hand*_, and the results of EC and VMHC were basically consistent with FC in the CTS. The EC revealed that the information output from the dominant hemisphere to the contralateral hemisphere was weakened.

**Conclusion:**

This study found that maladjusted connections between and within the bilateral S1 revealed by these methods are present in patients with CTS. The dominant hemisphere with deafferentation weakens its effect on the contralateral hemisphere. The disturbance in the bilateral S1 provides reliable evidence to understand the neuropathophysiological mechanisms of decreased functional integration in the brains of patients with CTS.

## Introduction

Interhemispheric and intrahemispheric long-range synchronization and information communication are crucial features of functional integration between the left and right hemispheres (Xinhu et al., 2020). Integration has been found to play an important role in multiple high-order functional processes, such as vision, attention, and sensory and motor functions (Antonello et al., 2016). Therefore, studies of various clinical diseases and neurophysiologic contexts, such as autism (Xiaonan et al., 2020), mild cognitive impairment (Li et al., 2021), chronic insomnia (Zhou F. et al., 2018), stroke (Lee et al., 2018), aging (Coelho et al., 2021), and the use of different gestures (Balconi and Fronda, 2021), have focused on these representative brain communication pathways to further understand the relevant pathophysiological processes.

Resting-state functional magnetic resonance imaging (rs-fMRI) has commonly been used to investigate brain connectivity between and within the two hemispheres (Biswal et al., 1995). Based on fMRI, functional connectivity (FC) and effective connectivity (EC) are two powerful methods to investigate the functional integration between different brain regions and reflect interactions across different cerebral regions (Fox and Raichle, 2007). By means of interhemispheric and intrahemispheric FC and EC, investigators could directly quantify the functional integration between and within the two brain hemispheres and thus have a considerable chance of determining how functional integration affects higher functional processing (Jin et al., 2020). FC, the statistical correlation between two or more brain regions, is considered a common approach to measuring non-directional interactions in the human brain (Zhou Y. et al., 2018; Reid et al., 2019). EC, such as the one shown by granger causality analysis (GCA), reveals the direction of the information flow by focusing on the time lag in the relationship between different brain regions (Xu et al., 2019). Different from the same time series of FC, GCA results are calculated in multiple time series (von Eye et al., 2014). However, both FC and EC have been used to identify the magnitude of functional connections between different brain regions in a variety of neurological and psychiatric disorders. Interestingly, different studies have shown multiple patterns of functional integration; for example, Coelho et al. (2021) have shown that aging induces decreased intrahemispheric connectivity and increased interhemispheric connectivity, which reflect a reduction in integration (Coelho et al., 2021). In contrast, patients with stroke showed disrupted interhemispheric connectivity (Lee et al., 2018). A study of temporary functional deafferentation also revealed that the interhemispheric FC of the sensorimotor area of healthy individuals was significantly reduced after peripheral nerve blockade, while the intrahemispheric FC changed inappreciably (Melton et al., 2016). Meanwhile, a similar pattern of interhemispheric plasticity has been demonstrated by fMRI in patients with different peripheral nerve injuries (PNIs) (Chemnitz et al., 2015; Chao et al., 2018). In addition, we also adopted voxel-mirrored homotopic connectivity (VMHC) to qualify the coordination of the primary somatosensory cortex (S1) in whole-brain interhemispheric FC, which was represented by the FC between each voxel in one hemisphere and its mirrored counterpart in the opposite hemisphere (Stark et al., 2008).

Normally, the experience of the external and internal environment is properly accepted as an input signal and accommodated by the cerebral cortex. PNIs disorder the daily function of the brain processes in manual behaviors, and functional and structural remapping continuously proceed in the entire cerebrum (Bhat et al., 2017; Onishi et al., 2018). The deafferented sensory input from the injured body part causes neural activity not only in the contralateral cortex but also in the ipsilateral, homotopic cortical area (Yu and Koretsky, 2014). The cortical projection territories of the corresponding afferent nerve are disordered and invaded from the adjacent area (Wu et al., 2018). In particular, the neuronal activity following PNI is changed in the contralateral and ipsilateral S1s, which are related to enduring symptoms of sensory dysfunction, such as paresthesia, numbness, pain, and weakness (Han et al., 2013). Individuals with carpal tunnel syndrome (CTS), a typical nerve entrapment PNI, exhibited a greater activated extent in the contralateral S1 and different ipsilateral activity during the activation task compared to healthy individuals (Nordmark and Johansson, 2020). Meanwhile, our previous study found increased intrahemispheric FC and decreased interhemispheric FC. We previously suggested that increased FC is supplementary to the afferent block, and decreased FC implies conduction damage of bilateral hemispheric information exchange (Lu et al., 2017).

We speculated that the decreased synaptic activity suppressed the synchronization effect from the contralateral hemisphere, but we still have doubts about the direction of these information-interaction effects. Previous studies used a relatively rough subarea template to clarify refined connectivity information, which may provide imprecise results, such as S1 spanning multiple functional regions. The Brainnetome is a finely sorted human brain atlas based on connectional architecture and links brain connectivity to function. It was considered the brain template that we adopted in this study to explore the strength of functional and directional interactions and the coordination between the bilateral S1 (Fan et al., 2016).

As described earlier, combined with our previous results (Lu et al., 2017), eight subregions of the bilateral S1 in the human Brainnetome Atlas were extracted as the regions of interest (ROIs) (Fan et al., 2016). The FC, GCA, and VMHC were adopted to explore the alternations of interhemispheric and intrahemispheric information communication of the S1. The directional and non-directional connectivity patterns between ROIs were computed to verify our hypothesis. We hypothesized that the interhemispheric and intrahemispheric integration would be changed in the patients with CTS, and the EC and FC results would mutually be corroborated. Therefore, this study investigated the changed connectivity of communication functions and coordination of central neural regions in patients with CTS.

## Materials and Methods

### Participants

All the data were acquired from 76 right-handed participants who were recruited from the Yueyang Hospital of Integrated Traditional Chinese and Western Medicine, Shanghai University of Traditional Chinese Medicine. Participants included 53 patients with bilateral CTS and 23 healthy control subjects (HCs). Written informed consent was obtained from each subject. This study was approved by the Medical Ethics Committee of Yueyang Hospital. All ethics-related work was performed in accordance with the Declaration of Helsinki.

The patients with CTS presented with a stage with objective neurological signs and delayed motor conduction. To ensure these criteria, one professional hand surgeon was involved throughout the diagnosis of all patients and healthy subjects. Inclusion criteria were as follows: (1) complaints of paresthesia/numbness in the median nerve innervated territories, night pain, wrist/finger weakness, and/or thenar atrophy in bilateral hands for more than 3 months according to the guideline released by Lu et al. (2017); (2) Phalen’s sign and Tinel’s sign; and (3) motor latency of the median nerve above 3.7 ms. The exclusion criteria for both groups include (1) confirmed or suspected history of peripheral neuropathies or cerebral diseases and (2) MRI contraindications.

### fMRI Data Acquisition

Each participant was instructed before scanning to remain at rest and awake without thinking or falling asleep. Matching hoods and foam pads were used to fix the head and reduce head motion. The images were acquired using a Magnetom Trio A 3T MR Scanner (Siemens AG, Erlangen, Germany). Rs-fMRI images were acquired using a gradient echo-echo planar imaging (GRE-EPI) sequence with the following parameters: interleaved scanning order; slice number = 43; matrix size = 64 × 64; field of view (FOV) = 240 mm × 240 mm; repetition time/echo time (TR/TE) = 3,000/30 ms; flip angle = 90°; slice thickness = 3.0 mm; acquisition voxel size = 3.2 mm × 3.2 mm × 3.40 mm; and number of repetitions = 240 for a total acquisition time of 12 min.

### fMRI Data Preprocessing

Data preprocessing procedures were performed using the Statistical Parametric Mapping 12 (SPM 12) toolbox^1^ based on the MATLAB 2014a platform. The first 10 volumes were removed to eliminate unstable signals. The subsequent preprocessing steps included slice timing, head motion correction, coregistration to individual anatomical images, spatial normalization to the EPI template of the Montreal Neurological Institute (MNI) space, resampling to 3.0 mm × 3.0 mm × 3.0 mm, and smoothing with a 6-mm full-width at half-maximum Gaussian kernel. Linear detrending and bandpass filtering (0.01–0.08 Hz) were further carried out. Finally, the nuisance signals, including the averaged signal from white matter, cerebrospinal fluid, and Friston 24 head motion parameters, were regressed out of the data. Five normal healthy subjects and three patients were abandoned because of excessive head motion (more than 2° and 2 mm) or serious artifacts. Finally, the images of the remaining 68 subjects were included in this study.

### Functional Connectivity and Extracted Regions of Interest

The main focus was on the S1 cortices, which were defined as the walls of the postcentral gyrus inside the central sulcus. To explore which connections contributed to alterations in patients with CTS, seed-based FC analyses were further conducted in eight regions of bilateral sub-S1 as ROIs, which were performed using the Resting-State fMRI Data Analysis Toolkit (REST) software^2^. The first pair of sub-S1 (PoG_L/R_1) regions represents the upper limb, head, and face, the second pair (PoG_L/R_2) represents the tongue and larynx, the fourth pair (PoG_L/R_4) is the trunk region, and the third pair (PoG_L/R_3) represents other parts of the body (Fan et al., 2016). Specifically, for each individual, the mean time series of each seed point was calculated by averaging the functional MRI time series for all voxels within each ROI and then correlating them with the time series of the rest of the whole brain in a voxelwise way using the preprocessed functional images. The resultant correlation maps were subsequently normalized with Fisher’s *r* to *Z* transformation.

### Granger Causality Analysis

In this study, we also used the REST toolbox to explore the causal interaction among the eight subregions (Zang et al., 2012). According to previous studies, GCA was based on multiple linear regressions and considered a credible method to investigate causal connectivity (Roebroeck et al., 2005). The GCA protocol was performed as follows (van Ettinger-Veenstra et al., 2019). We intended to explore the EC of all the sub-S1 regions. All the ROI coordinates were in the MNI space. ROI-wise GCA was performed using the selected ROIs. We used the RESTplus toolkit to perform the bivariate ROI-wise GCA pipeline. For each participant, the causal effects among the ROIs were analyzed. The alterations in EC were calculated by computing the bivariate coefficients between the patients and healthy controls.

### Voxel-Mirrored Homotopic Connectivity

The analysis of VMHC was also performed using REST software. For each subject, the homotopic FC was computed as the Pearson’s correlation coefficient between each voxel’s preprocessed signal time series and that of its symmetrical counterpart in the other hemisphere. Correlation coefficients were then Fisher’s *Z*-transformation to improve normality. The resultant *Z*-values, constituting the VMHC, were used for subsequent voxelwise group comparison.

### Statistical Analysis

A two-sample *t*-test was performed to contrast the results between CTS and HCs. Those considered significant results were passed the false discovery rate (FDR) correction (*p* < 0.05). Autoregression coefficients of ROI-wise GCA results between CTS and HCs were compared using the two-sample *t*-test based on the Social Sciences 21.0 (IBM SPSS Inc., United States).

## Results

### Functional Connectivity

Compared to the HCs, the patient group with CTS showed a significantly changed FC (FDR, *p* < 0.05).

**FIGURE 1:**
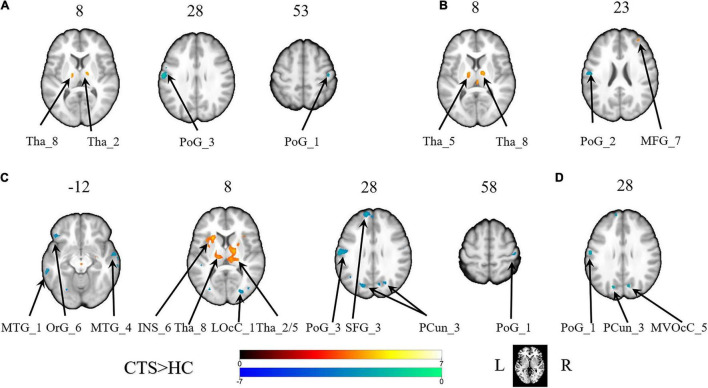
The brain regions with a significant difference in the FC of the sub-primary sensory cortex between patients with CTS and healthy controls. **(A)** The left first sub-S1. **(B)** The left third sub-S1. **(C)** The left second sub-S1. **(D)** The right second sub-S1. INS, insular gyrus; LOcC, lateral occipital cortex; MFG, middle frontal gyrus; MTG, middle temporal gyrus; MVOcC, MedioVentral occipital cortex; OrG, orbital gyrus; PCun, precuneus; PoG, postcentral gyrus; SFG, superior frontal gyrus; Tha, thalamus. L, left. R, right.

With the predefined four subregions in the left S1, increased FC was observed between the PoG_L_1 and the bilateral thalamus, the PoG_L_2 and the bilateral thalamus, the PoG_L_2 and the left insular, the PoG_L_3 and the right middle frontal gyrus, and the PoG_L_3 and the bilateral thalamus. Decreased FC was exhibited between the PoG_L_1 and the ipsilateral PoG_L_3, the PoG_L_1 and the contralateral PoG_R_1, the PoG_L_2 and bilateral middle temporal gyrus, bilateral precuneus, left superior frontal gyrus, left orbital gyrus, the ipsilateral PoG_L_1, the contralateral PoG_R_3, the right lateral occipital cortex, the PoG_L_3, and the ipsilateral PoG_L_2 (Figures 1A–C and Table 1).

**TABLE 1:** Regions showing significant differences in functional connectivity of the sub-S1 areas between patients with CTS and HCs (*p*_False discovery rate_ < 0.05).

Contrast Name					MNI Coordinates		
ROI-seed		Region label	Extent	*t*-value	X	y	z
PoG_L_1	Positive	Tha_L_8	21	4.7168	−18	−18	0
		Tha_R_2	13	4.6085	15	−6	6
	Negative	PoG_L_3	47	−6.1465	−60	−15	30
		PoG_R_1	12	−4.7988	48	−21	57
PoG_L_2	Positive	INS_L_6	50	5.4193	−33	0	15
		Tha_R_2	117	5.1319	15	−6	6
		Tha_R_5	117	4.4707	9	−30	3
		Tha_L_8	61	4.4567	−15	−18	9
	Negative	MTG_L_1	75	−5.9152	−63	−36	−6
		PoG_L_3	67	−4.8182	−57	−15	30
		MTG_R_4	42	−4.8052	63	−3	−15
		SFG_L_3	36	−4.7037	−12	57	27
		PoG_R_1	15	−4.5723	48	−21	57
		PCun_L_3	33	−4.5277	−18	−75	27
		PCun_R_3	20	−4.5268	21	−69	30
		OrG_L_6	17	−4.2139	−48	30	−12
		LOcC_R_1	21	−4.1363	30	−81	9
		MTG_R_1	10	−4.026	69	−24	−12
PoG_R_2	Negative	MVOcC_R_5	20	−5.348	9	−75	30
		PoG_L_1	40	−5.0868	−54	−6	21
		PCun_L_3	20	−4.8597	−18	−75	27
PoG_L_3	Positive	MFG_R_4	11	4.2102	36	51	21
		Tha_L_5	29	4.8051	−12	−9	9
		Tha_R_8	28	4.8348	12	−9	9
	Negative	PoG_L_2	35	−4.7809	−60	−15	27

*The corrected threshold of p < 0.05 was determined by Monte Carlo simulation. MNI, Montreal Neurological Institute; INS, Insular Gyrus; LOcC, lateral Occipital Cortex; MFG, Middle Frontal Gyrus; MTG, Middle Temporal Gyrus; MVOcC, MedioVentral Occipital Cortex; OrG, Orbital Gyrus; PCun, Precuneus; PoG, Postcentral Gyrus; SFG, Superior Frontal Gyrus; Tha, Thalamus. L, left: R, right.*

With the predefined four subregions in the right S1, decreased FC was displayed between the PoG_R_3 and the contralateral PoG_L_1, right temporal gyrus, and right medioventral occipital cortex (Figure 1D and Table 1).

There were no significant differences in the other sub-S1 regions.

### Effective Connectivity

**FIGURE 2:**
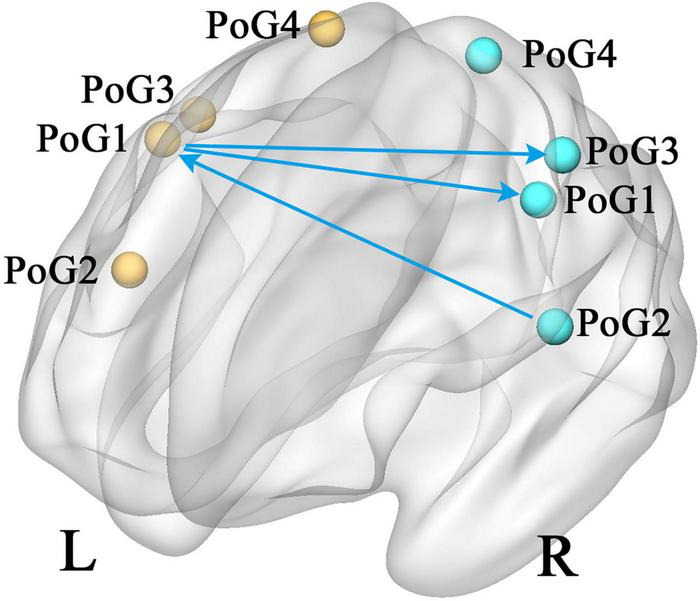
Group differences in effective connectivity of sub S1 (CTS > HCs, *p* < 0.05). PoG, postcentral gyrus; L, left; R, right.

According to the eight selected sub-S1 regions, the GCA analysis between each pair was computed. In contrast to the healthy controls, the patients with CTS demonstrated significantly decreased values from the PoG_R_1 to the contralateral PoG_L_1 and the right PoG_R_3, and from the PoG_R_2 to the contralateral PoG_L_1 (*p* < 0.05, Figure 2).

### Voxel-Mirrored Homotopic Connectivity

**FIGURE 3:**
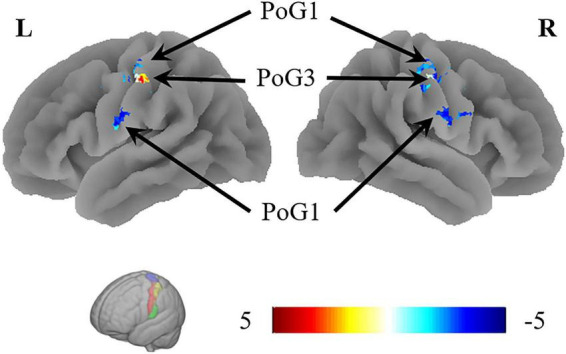
The results of VMHC revealed the coordination of the bilateral PoG_1 and PoG_3. L, left; R, right.

The VMHC results revealed that the coordination of the bilateral PoG_1 was decreased, while the coordination of the bilateral PoG_3 was increased (Figure 3 and Table 2).

**TABLE 2:** Regions showing significant differences in VMHC of the sub-S1 areas between patients with CTS and HCs.

Contrast Name				MNI Coordinates		
	Region Label	Extent	*t*-value	X	y	z
Positive	PoG_L_3	11	5.208	57	−24	48
	PoG_L_3	10	5.208	−57	−24	48
Negative	PoG_R_1	32	−5.038	51	−15	48
	PoG_L_1	32	−5.038	−51	−15	48
	PoG_R_1	26	−4.422	63	−6	24
	PoG_L_1	18	−4.422	−63	−6	24

*The corrected threshold of p < 0.001.*

## Discussion

In this study, the results of abnormal connective and coordination patterns between and within the bilateral S1 are shown by the FC, GCA, and VMHC results. In contrast to most previous studies of interhemispheric and intrahemispheric connections, we applied more precise brain regions to explore abnormally changed connective characteristics. In addition, more interhemispheric results were shown in this CTS study than in the intrahemispheric study, especially for EC and VMHC. We found significantly decreased strength of the two connectivities in the interhemispheric S1. The information output from the dominant hemisphere to the contralateral hemisphere was weakened. Meanwhile, FC displayed a significant intrahemispheric decrease. The results of EC regarding the strength of connection were basically consistent with those of FC. Similarly, the VMHV value of the hand representative region (S1_*hand*_), PoG_4_1, also decreased. These results revealed that the information communication efficiency between the two hemispheres was obstructive.

Numerous studies have examined the changeable relationship in the interhemispheric and intrahemispheric FC of integration. In a study on stroke, Baldassarre et al. reported that focal brain lesions induce a reduction in interhemispheric FC and an increase in intrahemispheric FC^2^. Liu et al. (2015) examined the effects of lesions and treatment-based recovery on functional organization and found that increased interhemispheric FC between the bilateral primary motor cortex (M1) was positively correlated with motor function recovery. Therefore, FC was thought to be a marker to predict behavioral deficits after stroke. Meanwhile, a similar integration pattern has been demonstrated not only in stroke but also in different diseases (Chen and Schlaug, 2013; Lee et al., 2018). Impaired and reduced white matter fibers have been found in children with cerebral palsy (Weinstein et al., 2014). In a study of schizophrenia, investigators found that the disconnection between brain hemispheres represents a derailment of cognitive functions. In addition, the asymmetry of hemispheric network properties was associated with patients’ symptom severity, such as the severity of hallucinations and delusions, which showed an increase with increasing interhemispheric connectivity in the right frontal and bilateral temporal cortices (Zhang et al., 2019). Other research on diseases, including dementia (Filippi et al., 2017), epilepsy (Hung et al., 2019), and acute damage to the corpus callosum (Ridley et al., 2016), among others, also reported a disordered integration pattern. Interestingly, connectivity also changed even in healthy subjects. Duncan et al. demonstrated that the differential processing demands of two scripts influenced both interhemispheric and intrahemispheric interactions; compared to Hiragana, Kanji could increase activation in right hemisphere areas or within a ventral visual form-to-meaning pathway and increase interhemispheric connectivity.

In contrast to central nervous diseases, in PNIs, the brain is complete and undamaged. Compared to healthy subjects, long-term aberrant signal inputs changed the corresponding cortical representation of the injured nerve (Irvine and Rajan, 1996). Recent studies have indicated that denervation always causes reorganization in the bilateral sensory cortex. For instance, research on a rodent whisker system has confirmed that trimming whiskers in mice could lead to loss of synaptic connections in S1 and change sensorimotor integration (Barnes and Finnerty, 2010). Persistent hand representation still exists in the S1 of amputees after denervation, and activation was found in the corresponding sensory areas when the amputees attempted to move their phantom limb (Irvine and Rajan, 1996). Elevated gray matter volume was found in the S1 of patients with chronic low back pain (Kim et al., 2020). This study also found that the alterations were not limited to and even overstepped the cortical representation of the body area to which persistent pain was attributed. Similarly, in CTS, a previous study suggested that sensory afferent interruption induces a decline in synaptic activity in S1 (Lu et al., 2017). In contrast, another study also indicated that cortical plasticity expands from the S1-hand area to the S1-leg and S1-face areas (Maeda et al., 2017). Functional cortical remapping occurred in distinctly defined subregions of ipsilateral S1 after CTS. Therefore, the interactions in S1 may be the consequence of an internal compensatory response.

Interestingly, a previous study reported that the connectivity between the areas located far apart from each other was vulnerable, such as the heterotopic interhemispheric structural connections in the sensorimotor network (Straathof et al., 2020). A human study also supports the opinion that, compared to the other high-order association regions, the primary sensory-motor cortices demonstrated relatively lower functional stability during resting-state scans (Li et al., 2020). The instability reflects both external stimuli and top-down modulation from high-order regions (Macaluso and Driver, 2005). Li et al. (2020) found that the bilateral primary visual cortices displayed lower stability during the viewing task than in the resting state. They deduced that sensory inputs directly affect the neural activity of visual cortices, and the decreased stability could be caused by the continuous reorganization of functional architecture to changes in the received visual form over time. Similarly, patients with CTS always suffered sustained abnormal stimuli. Based on Li’s inference, a possible interpretation of our results was that continuous stimuli and modulation would worsen the frail connection connecting the bilateral S1. The weakened VMHC results further suggested that coordination of the bilateral hand representative brain area was impaired after CTS. The increased VMHC confirmed that compensatory remodeling took place in the other sub-S1 region. Meanwhile, the EC showed that, compared with the HCs, the output information flow from the dominant S1_*hand*_ of patients with CTS was prominently decreased. The series of consequences demonstrated that aberrant and persistent sensory stimulation from the dominant hand weakened the strength of the output information of the contralateral S1_*hand*_ and decreased the connectivity between the S1_*hand*_ and other brain regions directly.

In addition, our FC results offer an accordant and interesting phenomenon, namely, the connections between the sub-S1s and thalamus were activated. Generally, the sensory processing pathways in the cerebral cortex occur not only *via* direct communication between the primary and secondary sensory areas but also *via* a parallel transthalamic circuit (Mo and Sherman, 2019). Robust and effective synaptic connections between the S1 projections to the thalamus have been confirmed by *trans*-synaptic tracing, and the latter carries information to other cortical areas (Mo and Sherman, 2019). Animal studies also reported that transection of the sensory nerve could lead to an increasing number of afferent fibers to the thalamic neurons (Takeuchi et al., 2012). This evidence demonstrates our result that CTS could activate the pathway from S1 to the thalamus.

Several limitations should be declared. First, this cross-sectional study does not provide direct evidence of a correlation between FC and the clinical assessment, similar to EC. Future studies using different scales are needed to deepen our understanding of the neural mechanisms underlying CTS. Second, the FC and GCA analyses were based on the whole brain. However, the somatosensory network, the specific functional resting-state network related to the motor-sensory network, should be regarded as the anatomical substrate to analyze sensory connectivity in future studies. Third, the GCA method has been debated due to the effects of hemodynamic convolution; therefore, the dynamic causal model, which can compensate for the defects of the GCA, could be used to provide accurate results for future research (Friston, 2011).

## Conclusion

In this study, we demonstrated that maladjusted connections between and within the bilateral S1 revealed by FC and EC are present in patients with CTS. The patients with CTS showed decreased FC between the bilateral sub-S1 regions and increased FC between the sub-S1 regions and thalamus. Meanwhile, the decreased VMHC and causal information flow from the advantaged S1_*hand*_ to the disadvantaged S1_*hand*_ were consistent with the FC results to some extent and reflected that the prominent cortices are more easily influenced by the abnormal information from the corresponding afferent nerve. The disturbance in the bilateral sub-S1 will provide reliable evidence to understand the neuropathophysiological mechanisms in patients with CTS.

## Data Availability Statement

The raw data supporting the conclusions of this article will be made available by the authors, without undue reservation.

## Ethics Statement

The studies involving human participants were reviewed and approved by Medical Ethics Committee of Yueyang hospital. The patients/participants provided their written informed consent to participate in this study.

## Author Contributions

X-XX and X-YH: conceptualization and writing–original draft. X-YH, M-XZ, and J-JW: methodology. JM, Z-ZM, and Y-JD: validation. X-XX and Z-ZM: formal analysis. J-GX: writing, review and editing. All authors read and approved the final manuscript.

## Conflict of Interest

The authors declare that the research was conducted in the absence of any commercial or financial relationships that could be construed as a potential conflict of interest.

## Publisher’s Note

All claims expressed in this article are solely those of the authors and do not necessarily represent those of their affiliated organizations, or those of the publisher, the editors and the reviewers. Any product that may be evaluated in this article, or claim that may be made by its manufacturer, is not guaranteed or endorsed by the publisher.

## Footnotes

1
http://www.fil.ion.ucl.ac.uk/spm/

2
http://www.restfmri.net/forum/

## Abbreviations

CTS: carpal tunnel syndrome
EC: effective connectivity
FC: functional connectivity
S1: primary somatosensory cortex
PNI: peripheral nerve injury
rs-fMRI: resting-state functional magnetic resonance
GCA: granger causality analysis
ROIs: regions of interest
HCs: healthy control subjects
MNI: Montreal Neurological Institute
VMHC: voxel-mirrored homotopic connectivity.

## References

[B1] Antonello B. Rl E. Sj S. Sg L. Maurizio C. (2016). Brain connectivity and neurological disorders after stroke. *Curr. Opin. Neurol.* 29, 706–713. 10.1097/WCO.0000000000000396 27749394 PMC5682022

[B2] Balconi M. Fronda G. (2021). Intra-Brain Connectivity vs. Inter-Brain Connectivity in Gestures Reproduction: what Relationship? *Brain Sci.* 11:577. 10.3390/brainsci11050577 33947101 PMC8145238

[B3] Barnes S. J. Finnerty G. T. (2010). Sensory experience and cortical rewiring. *Neuroscientist* 16 186–198. 10.1177/1073858409343961 19801372

[B4] Bhat D. I. Indira D. B. Bharti K. Panda R. (2017). Cortical plasticity after brachial plexus injury and repair: a resting-state functional MRI study. *Neurosurg. Focus* 42:E14. 10.3171/2016.12.FOCUS16430 28245732

[B5] Biswal B. Yetkin F. Z. Haughton V. M. Hyde J. S. (1995). Functional connectivity in the motor cortex of resting human brain using echo-planar MRI. *Magn. Reson. Med.* 34 537–541. 10.1002/mrm.1910340409 8524021

[B6] Chao T. H. Chen J. H. Yen C. T. (2018). Plasticity changes in forebrain activity and functional connectivity during neuropathic pain development in rats with sciatic spared nerve injury. *Mol. Brain* 11:55. 10.1186/s13041-018-0398-z 30285801 PMC6167811

[B7] Chemnitz A. Weibull A. Rosén B. Andersson G. Dahlin L. B. Björkman A. (2015). Normalized activation in the somatosensory cortex 30 years following nerve repair in children: an fMRI study. *Eur. J. Neurosci.* 42 2022–2027. 10.1111/ejn.12917 25865600

[B8] Chen J. L. Schlaug G. (2013). Resting state interhemispheric motor connectivity and white matter integrity correlate with motor impairment in chronic stroke. *Front. Neurol.* 4:178. 10.3389/fneur.2013.00178 24223571 PMC3819700

[B9] Coelho A. Fernandes H. M. Magalhães R. Moreira P. S. Marques P. Soares J. M. (2021). Reorganization of brain structural networks in aging: a longitudinal study. *J. Neurosci. Res.* 99 1354–1376. 10.1002/jnr.24795 33527512 PMC8248023

[B10] Fan L. Li H. Zhuo J. Zhang Y. Wang J. Chen L. (2016). The Human Brainnetome Atlas: a New Brain Atlas Based on Connectional Architecture. *Cereb. Cortex* 26 3508–3526. 10.1093/cercor/bhw157 27230218 PMC4961028

[B11] Filippi M. Basaia S. Canu E. Imperiale F. Meani A. Caso F. (2017). Brain network connectivity differs in early-onset neurodegenerative dementia. *Neurology* 89 1764–1772. 10.1212/WNL.0000000000004577 28954876 PMC5664301

[B12] Fox M. D. Raichle M. E. (2007). Spontaneous fluctuations in brain activity observed with functional magnetic resonance imaging.. *Nat Rev. Neurosci.* 8 700–711. 10.1038/nrn2201 17704812

[B13] Friston K. J. (2011). Functional and effective connectivity: a review. *Brain Connect* 1 13–36. 10.1089/brain.2011.0008 22432952

[B14] Han Y. Li N. Zeiler S. R. Pelled G. (2013). Peripheral nerve injury induces immediate increases in layer v neuronal activity. *Neurorehabil. Neural. Repair.* 27 664–672. 10.1177/1545968313484811 23599222 PMC3729632

[B15] Hung S. C. Lee C. C. Chen H. H. Chen C. Wu H. M. Lin C. P. (2019). Early recovery of interhemispheric functional connectivity after corpus callosotomy. *Epilepsia* 60 1126–1136. 10.1111/epi.14933 31087658

[B16] Irvine D. R. Rajan R. (1996). Injury- and use-related plasticity in the primary sensory cortex of adult mammals: possible relationship to perceptual learning. *Clin. Exp. Pharmacol. Physiol.* 23 939–947. 10.1111/j.1440-1681.1996.tb01146.x 8911738

[B17] Jin X. Liang X. Gong G. (2020). Functional Integration Between the Two Brain Hemispheres: evidence From the Homotopic Functional Connectivity Under Resting State. *Front. Neurosci.* 14:932. 10.3389/fnins.2020.00932 33122984 PMC7566168

[B18] Kim H. Mawla I. Lee J. Gerber J. Walker K. Kim J. (2020). Reduced tactile acuity in chronic low back pain is linked with structural neuroplasticity in primary somatosensory cortex and is modulated by acupuncture therapy. *Neuroimage* 217:116899. 10.1016/j.neuroimage.2020.116899 32380138 PMC7395964

[B19] Lee J. Park E. Lee A. Chang W. H. Kim D. S. Kim Y. H. (2018). Alteration and Role of Interhemispheric and Intrahemispheric Connectivity in Motor Network After Stroke. *Brain Topogr.* 31 708–719. 10.1007/s10548-018-0644-9 29671156

[B20] Li L. Lu B. Yan C. G. (2020). Stability of dynamic functional architecture differs between brain networks and states. *Neuroimage* 216:116230. 10.1016/j.neuroimage.2019.116230 31577959

[B21] Li W. Xu X. Wang Z. Peng L. Wang P. Gao X. (2021). Multiple Connection Pattern Combination From Single-Mode Data for Mild Cognitive Impairment Identification. *Front. Cell. Dev. Biol.* 9:782727. 10.3389/fcell.2021.782727 34881247 PMC8645991

[B22] Liu J. Qin W. Zhang J. Zhang X. Yu C. (2015). Enhanced interhemispheric functional connectivity compensates for anatomical connection damages in subcortical stroke. *Stroke* 46 1045–1051. 10.1161/STROKEAHA.114.007044 25721013

[B23] Lu Y. C. Zhang H. Zheng M. X. Hua X. Y. Qiu Y. Q. Shen Y. D. (2017). Local and extensive neuroplasticity in carpal tunnel syndrome: a resting-state fMRI study. *Neurorehabil. Neural. Repair* 31 898–909. 10.1177/1545968317723749 28845734

[B24] Macaluso E. Driver J. (2005). Multisensory spatial interactions: a window onto functional integration in the human brain. *Trends Neurosci.* 28 264–271. 10.1016/j.tins.2005.03.008 15866201

[B25] Maeda Y. Kim H. Kettner N. Kim J. Cina S. Malatesta C. (2017). Rewiring the primary somatosensory cortex in carpal tunnel syndrome with acupuncture. *Brain* 140 914–927. 10.1093/brain/awx015 28334999 PMC5837382

[B26] Melton M. S. Browndyke J. N. Harshbarger T. B. Madden D. J. Nielsen K. C. Klein S. M. (2016). Changes in Brain Resting-state Functional Connectivity Associated with Peripheral Nerve Block: a Pilot Study. *Anesthesiology* 125 368–377. 10.1097/ALN.0000000000001198 27272674 PMC4955751

[B27] Mo C. Sherman S. M. A. (2019). Sensorimotor Pathway via Higher-Order Thalamus. *J. Neurosci.* 39 692–704. 10.1523/JNEUROSCI.1467-18.2018 30504278 PMC6343647

[B28] Nordmark P. F. Johansson R. S. (2020). Disinhibition of Human Primary Somatosensory Cortex After Median Nerve Transection and Reinnervation. *Front. Hum. Neurosci.* 14:166. 10.3389/fnhum.2020.00166 32499687 PMC7242759

[B29] Onishi O. Ikoma K. Oda R. Yamazaki T. Fujiwara H. Yamada S. (2018). Sequential variation in brain functional magnetic resonance imaging after peripheral nerve injury: a rat study. *Neurosci. Lett.* 673 150–156. 10.1016/j.neulet.2018.03.003 29524643

[B30] Reid A. T. Headley D. B. Mill R. D. Sanchez-Romero R. Uddin L. Q. Marinazzo D. (2019). Advancing functional connectivity research from association to causation. *Nat. Neurosci.* 22 1751–1760. 10.1038/s41593-019-0510-4 31611705 PMC7289187

[B31] Ridley B. Beltramone M. Wirsich J. Le Troter A. Tramoni E. Aubert S. (2016). Alien Hand, Restless Brain: salience Network and Interhemispheric Connectivity Disruption Parallel Emergence and Extinction of Diagonistic Dyspraxia. *Front. Hum. Neurosci.* 10:307. 10.3389/fnhum.2016.00307 27378896 PMC4913492

[B32] Roebroeck A. Formisano E. Goebel R. (2005). Mapping directed influence over the brain using Granger causality and fMRI. *Neuroimage* 25 230–242. 10.1016/j.neuroimage.2004.11.017 15734358

[B33] Stark D. E. Margulies D. S. Shehzad Z. E. Reiss P. Kelly A. M. Uddin L. Q. (2008). Regional variation in interhemispheric coordination of intrinsic hemodynamic fluctuations. *J. Neurosci.* 28 13754–13764. 10.1523/JNEUROSCI.4544-08.2008 19091966 PMC4113425

[B34] Straathof M. Sinke M. Roelofs T. Blezer E. Sarabdjitsingh R. A. van der Toorn A. (2020). Distinct structure-function relationships across cortical regions and connectivity scales in the rat brain. *Sci. Rep.* 10:56. 10.1038/s41598-019-56834-9 31919379 PMC6952407

[B35] Takeuchi Y. Yamasaki M. Nagumo Y. Imoto K. Watanabe M. Miyata M. (2012). Rewiring of afferent fibers in the somatosensory thalamus of mice caused by peripheral sensory nerve transection. *J. Neurosci.* 32 6917–6930. 10.1523/JNEUROSCI.5008-11.2012 22593060 PMC6622207

[B36] van Ettinger-Veenstra H. Lundberg P. Alföldi P. Södermark M. Graven-Nielsen T. Sjörs A. (2019). Chronic widespread pain patients show disrupted cortical connectivity in default mode and salience networks, modulated by pain sensitivity. *J. Pain Res.* 12 1743–1755. 10.2147/JPR.S189443 31213886 PMC6549756

[B37] von Eye A. Wiedermann W. Mun E. Y. (2014). Granger causality–statistical analysis under a configural perspective. *Integr. Psychol. Behav. Sci.* 48 79–99. 10.1007/s12124-013-9243-1 23955018

[B38] Weinstein M. Green D. Geva R. Schertz M. Fattal-Valevski A. Artzi M. (2014). Interhemispheric and intrahemispheric connectivity and manual skills in children with unilateral cerebral palsy. *Brain Struct. Funct.* 219 1025–1040. 10.1007/s00429-013-0551-5 23571779

[B39] Wu J. J. Lu Y. C. Hua X. Y. Ma S. J. Xu J. G. A. (2018). Longitudinal Mapping Study on Cortical Plasticity of Peripheral Nerve Injury Treated by Direct Anastomosis and Electroacupuncture in Rats. *World Neurosurg.* 114 e267–e282. 10.1016/j.wneu.2018.02.173 29524702

[B40] Xiaonan G. Xujun D. Heng C. Changchun H. Jinming X. Shaoqiang H. (2020). Altered inter- and intrahemispheric functional connectivity dynamics in autistic children. *Hum. Brain Mapp.* 41 419–428. 10.1002/hbm.24812 31600014 PMC7268059

[B41] Xinhu J. Xinyu L. Gaolang G. (2020). Functional Integration Between the Two Brain Hemispheres: evidence From the Homotopic Functional Connectivity Under Resting State. *Front. Neurosci.* 14:932. 10.3389/fnins.2020.00932 PMC7566168 33122984

[B42] Xu X. M. Jiao Y. Tang T. Y. Lu C. Q. Zhang J. Salvi R. (2019). Altered Spatial and Temporal Brain Connectivity in the Salience Network of Sensorineural Hearing Loss and Tinnitus. *Front. Neurosci.* 13:246. 10.3389/fnins.2019.00246 30941010 PMC6433888

[B43] Yu X. Koretsky A. P. (2014). Interhemispheric plasticity protects the deafferented somatosensory cortex from functional takeover after nerve injury. *Brain Connect.* 4 709–717. 10.1089/brain.2014.0259 25117691 PMC4238258

[B44] Zang Z. X. Yan C. G. Dong Z. Y. Huang J. Zang Y. F. (2012). Granger causality analysis implementation on MATLAB: a graphic user interface toolkit for fMRI data processing. *J. Neurosci. Methods* 203 418–426. 10.1016/j.jneumeth.2011.10.006 22020117

[B45] Zhang Y. Dai Z. Chen Y. Sim K. Sun Y. Yu R. (2019). Altered intra- and inter-hemispheric functional dysconnectivity in schizophrenia. *Brain Imaging Behav.* 13 1220–1235. 10.1007/s11682-018-9935-8 30094555

[B46] Zhou F. Zhao Y. Huang M. Zeng X. Wang B. Gong H. (2018). Disrupted interhemispheric functional connectivity in chronic insomnia disorder: a resting-state fMRI study. *Neuropsychiatr. Dis. Treat* 14 1229–1240. 10.2147/NDT.S162325 29795981 PMC5957476

[B47] Zhou Y. Qiao L. Li W. Zhang L. Shen D. (2018). Simultaneous Estimation of Low- and High-Order Functional Connectivity for Identifying Mild Cognitive Impairment. *Front. Neuroinform.* 12:3. 10.3389/fninf.2018.00003 29467643 PMC5808180

